# Slc:Wistar/ST rats develop unilateral thyroid dysgenesis: A novel animal model of thyroid hemiagenesis

**DOI:** 10.1371/journal.pone.0221939

**Published:** 2019-08-29

**Authors:** Teppei Nakamura, Osamu Ichii, Yuji Sunden, Yaser Hosny Ali Elewa, Tomoji Yoshiyasu, Hideki Hattori, Osamu Tatsumi, Yasuhiro Kon, Ken-ichi Nagasaki

**Affiliations:** 1 Section of Biomedical Science, Chitose Laboratory, Japan Food Research Laboratories, Chitose, Hokkaido, Japan; 2 Laboratory of Anatomy, Department of Basic Veterinary Sciences, Division of Veterinary Medicine, Faculty of Veterinary Medicine, Hokkaido University, Sapporo, Hokkaido, Japan; 3 Laboratory of Veterinary Pathology, Faculty of Agriculture, Tottori University, Tottori, Japan; 4 Department of Histology and Cytology, Faculty of Veterinary Medicine, Zagazig University, Zagazig, Egypt; 5 Section of Biological Safety Research, Chitose Laboratory, Japan Food Research Laboratories, Chitose, Hokkaido, Japan; 6 Section of Biological Safety Research, Tama Laboratory, Japan Food Research Laboratories, Tama, Tokyo, Japan; Laboratoire de Biologie du Développement de Villefranche-sur-Mer, FRANCE

## Abstract

Developmental anomalies of the thyroid gland lead to congenital malformations such as thyroglossal duct cysts and thyroid dysgenesis. However, the pathogenesis of thyroid dysgenesis remains unclear due to the lack of suitable animal models. This study demonstrated that Slc:Wistar/ST rats frequently developed unilateral thyroid dysgenesis, including hemiagenesis, characterized by the absence of one lobe. In Wistar/ST rats, each thyroid lobe was frequently different in size, and approximately 27% and 20% of the rats presented with hemihypoplasia and hemiagenesis of the thyroid gland, respectively. Dysgenesis was predominant on the left side in both sexes, without sex differences. At a young age, thyroid hemiagenesis did not alter body weight. In rats of both sexes with thyroid hemiagenesis, plasma total triiodothyronine and total triiodothyronine levels remained unchanged while plasma thyroid-stimulating hormone levels were significantly elevated in young rats. The remaining thyroid lobes increased in weight, but the follicular epithelial cells appeared normal in terms of their height and proliferating activities. On the side of thyroid dysgenesis, the parathyroid glands were normally localized and were situated at the same location as the contralateral glands. The ultimobranchial body remnants were localized at the level of the thyroid gland along with the cranial thyroid artery and vein, forming cell clusters or cystic structures and containing calcitonin-positive C-cells. In conclusion, Wistar/ST rats developed unilateral thyroid dysgenesis and may be novel and useful animal models for thyroid hemiagenesis in humans and for morphogenesis of pharyngeal pouch-derived organs.

## Introduction

In mammals, the thyroid gland contains two important endocrine cell types that have a different embryological origin [1, 2]. The follicular epithelial cells develop from the thyroid primordium, which bud from the foregut endoderm, descend in front of the foregut tube, reach a final position in front of the larynx and proximal trachea, and become bilobulated thyroid lobes [1]. In mammals, C-cells originate from the ultimobranchial body budding from the fourth pharyngeal pouch [2]. The ultimobranchial body migrates caudally, fuses to the thyroid primordium, and eventually differentiates into C-cells [2]. In addition to the ultimobranchial body, the fourth pharyngeal pouch also gives rise to the superior parathyroid glands [2]. The superior parathyroid glands are embedded within the thyroid gland but are separated by connective tissues [3]. Alternatively, the inferior parathyroid glands, originating from the third pharyngeal pouch, are embedded within the thyroid glands of rodents such as mice, rats, and hamsters that lack superior parathyroid glands [2].

If the organogenesis of the thyroid gland is anomalous, congenital malformations can occur such as thyroglossal duct cysts and thyroid dysgenesis [4, 5]. Thyroid dysgenesis describes a heterogeneous group of thyroid malformations, and the most prevalent conditions are ectopic thyroids, athyreosis, and hypoplasia [1]. Thyroid dysgenesis accounts for 85% of primary congenital hypothyroidism and is therefore clinically important [5]. Although genes responsible for thyroid dysgenesis have been identified, such as *Hhex*, *Foxe1*, *Nkx2-1*, *Pax-8*, *Tbx1*, and *Shh*, these genes account for only 2% of the malformations in humans; furthermore, their pathogenesis is not fully understood [6, 7]. Unlike other thyroid dysgenesis, thyroid hemiagenesis is characterized by the absence of one lobe and does not cause clinical symptoms in itself [1]. Thyroid hemiagenesis is a rare thyroid dysgenesis, and it has been reported that its prevalence ranges from 0.05% to 0.2% in healthy subjects, and it occurs mainly on the left side [1]. In healthy subjects, the female-to-male ratio of thyroid hemiagenesis has been estimated to be 1.3:1 without sex difference [8–11]. Although several reports demonstrate that thyroid hemiagenesis is predominantly found in females during thyroid examinations, noting a 3:1 to 7:1 female-to-male ratio, this trend is likely due to thyroid diseases generally being more predominant in females; therefore, a higher frequency of thyroid examinations for female patients is necessary [8, 12]. However, the pathogenesis of such anomalies remains unclear because of the lack of suitable animal models.

Wistar rats are albino and outbred, originating from the Wistar Institute (PA, USA). Wistar Institute-derived rats are sourced and available from many breeders worldwide, and are widely used in biomedical research. Wistar rats have different characteristics, such as genetic variations and distinct physiological functions, attributable to their specific breed [13], and background data regarding each Wistar strain are important for obtaining accurate results in toxicological and pharmaceutical studies. In our study, we found that Slc:Wistar/ST rats available from Japan SLC, Inc. (Shizuoka, Japan) frequently possessed thyroid dysgenesis, and we examined the prevalence of this condition as well as the histopathology of their thyroid glands.

## Materials and methods

### Animals

All procedures performed in studies involving animals were in accordance with the ethical standards of the Committee for Animal Experiments Ethics of Japan Food Research Laboratories. The protocol was approved by the Committee for Animal Experiments Ethics of Japan Food Research Laboratories (approval no. 20141211). Wistar/ST rats were purchased from Japan SLC, and BrlHan:WIST@Jcl (GALAS) rats were purchased from CLEA Japan (Tokyo, Japan). The rats were kept under deep anesthesia using isoflurane, and heparinized blood was collected from the caudal vena cava. They were euthanized by cutting the abdominal aorta. Each lobe of the thyroid gland was collected by cutting the isthmus at the midline, and each lobe was weighed. The relative weight of the total thyroid gland and large thyroid lobe was calculated by dividing body weight from both lobes and the large lobe of the thyroid gland, respectively. In addition, we selected two each of male and female Wistar/ST rats lacking a unilateral thyroid lobe by opening the front of their neck under anesthesia with sodium pentobarbital and analgesia with medetomidine hydrochloride. The two pairs of rats were mated, and the rats delivered once. The F1 litters were also examined for the thyroid lobe weight at 9 to 10 weeks of age as described above.

### Measurement of plasma hormone levels

The collected heparinized blood was centrifuged to obtain plasma. The plasma was used for measurement of thyroid-stimulating hormone (TSH), total triiodothyronine (T3), and total thyroxin (T4) using an enzyme-linked immunosorbent assay (ELISA). A characterization of the ELISA performance is listed in Table 1.

**Table 1:** Summary of ELISA performance.

Hormone	Kit	Cross reactivity	Sensitivity	CV
Thyroid-stimulating hormone	Rat TSH ELISA Kit (cat. #: MBS726442, MyBioSource, CA, USA)	Specific for TSH	0.1 ng/ml	<10%
Total triiodothyronine	Mouse/Rat T3, Total ELISA kit (cat. #: T3043T-100, Calbiotech, CA, USA)	Not reported	0.25 ng/ml	<10%^1^^)^
Total thyroxin	Mouse/Rat T4, Total ELISA kit (cat. #: T4044T-100, Calbiotech)	Not reported	1 μg/dl	<10%^1^^)^

1) The value is calculated by the standard samples, since it is not reported by the manufacturer.

### Histopathological analysis

For histopathological analysis, the laryngopharynx, including the thyroid gland, was fixed with 10% neutral buffered formalin and was embedded in paraffin. The sections were stained with hematoxylin and eosin (HE) or periodic acid-Schiff (PAS). Immunohistochemistry for proliferating cell nuclear antigen (PCNA, for proliferating cells), calcitonin (for C-cells), p63, pan-cytokeratin, galectin-3, Bcl-2, Nkx2-1 (for ultimobranchial body remnants), and Iba1 (for macrophages) was performed [14, 15]. The details of the procedures are listed in Table 2. The sections were deparaffinized, heated with Histo VTone (Nacalai tesque, Kyoto, Japan) for 30 minutes at 90°C, treated with 0.3% hydrogen peroxidase/methanol solution for 30 minutes to eliminate endogenous peroxidase, blocked with a blocking reagent, and incubated overnight with the primary antibodies at 4°C. Next, the sections were treated with the secondary antibodies for 30 minutes, followed by treatment with streptavidin-peroxidase (cat. #: 426061, Nichirei, Tokyo, Japan) for 30 minutes at approximately 25°C. The immunopositive reactions were developed using a 3,3'-diaminobenzidine tetrahydrochloride-H_2_O_2_ solution. The sections were then counterstained with hematoxylin. In immunofluorescence for CD3 (for T-cell) and CD79a (for B-cell), the sections were deparaffinized, heated with Histo VTone (Nacalai tesque) for 30 minutes at 90°C, blocked with a blocking reagent, and incubated overnight with the primary antibodies at 4°C. Next, the sections were treated with the secondary antibodies for 30 minutes at approximately 25°C, and coverslipped with DAPI Fluoromount-G (Southern Biotech, AL, USA). The sections were observed using an all-in-one fluorescence microscope (BZ-X800, Keyence, Osaka, Japan).

**Table 2:** Summary of immunostaining conditions.

Antigen	Blocking reagent	Primary antibody	Secondary antibody
Proliferating cell nuclear antigen (PCNA)	10% normal rabbit serum (cat. #: 426052, Nichirei, Tokyo, Japan)	Goat polyclonal (cat. #: sc-9857, 1:2000, Santa Cruz, TX, USA)	Biotinylated rabbit anti-goat IgG (cat. #: 416022, prediluted, Nichirei)
Calcitonin	10% normal goat serum (cat. #: 426042, Nichirei)	Rabbit polyclonal (cat. #: IS515, prediluted, DAKO, Glostrup, Denmark)	Biotinylated goat anti-rabbit IgG (cat. #: 426012, prediluted, Nichirei)
Pan-cytokeratin	10% normal rabbit serum (cat. #: 426052, Nichirei)	Mouse monoclonal (clone AE1/AE3, 1:100, DAKO)	Biotinylated rabbit anti-mouse IgG+IgA+IgM (cat. #: 426032, prediluted, Nichirei)
p63	10% normal rabbit serum (cat. #: 426052, Nichirei)	Mouse monoclonal (clone 4A4, prediluted, Nichirei)	Biotinylated rabbit anti-mouse IgG+IgA+IgM (cat. #: 426032, prediluted, Nichirei)
Galectin-3	10% normal goat serum (cat. #: 426042, Nichirei)	Rabbit polyclonal (cat. #: bs-0721R, 1:300, Bioss, MA, USA)	Biotinylated goat anti-rabbit IgG (cat. #: 426012, prediluted, Nichirei)
Bcl-2	10% normal goat serum (cat. #: 426042, Nichirei)	Rabbit polyclonal (cat. #: sc-492, 1:200, Santa Cruz)	Biotinylated goat anti-rabbit IgG (cat. #: 426012, prediluted, Nichirei)
Nkx2-1	10% normal rabbit serum (cat. #: 426052, Nichirei)	Mouse monoclonal (clone SPT24, prediluted, Nichirei)	Biotinylated rabbit anti-mouse IgG+IgA+IgM (cat. #: 426032, prediluted, Nichirei)
Iba1	10% Normal goat serum (cat. #: 42604w2, Nichirei)	Rabbit polyclonal (cat. #: 019–19741, Fujifilm Wako, Osaka, Japan)	Biotinylated goat anti-rabbit IgG (cat. #: 426012, prediluted, Nichirei)
CD3	2.5% normal horse serum (cat. #: DK-8828, Vector, CA, USA)	Rabbit monoclonal (clone SP7, 1:100, Nichirei)	DyLight 594 conjugated horse anti-rabbit IgG (cat. #: DK-8828, Vector)
CD79a	2.5% normal horse serum (cat. #: DK-8828, Vector)	Mouse monoclonal (clone HM57, 1:200, Novus, CO, USA)	DyLight 488 conjugated horse anti-mouse IgG (cat. #: DK-8828, Vector)

### Statistical analysis

The results are expressed as mean ± standard error or box plot. The Mann-Whitney *U* test was used to compare data between two groups. The Kruskal-Wallis test was used to compare data among three groups, and multiple comparisons were performed using Scheffé’s method when a significant difference was noted. The categorical data were analyzed using the Fisher's exact test.

## Results

### Unilateral thyroid dysgenesis in Wistar/ST rats

**Fig 1:**
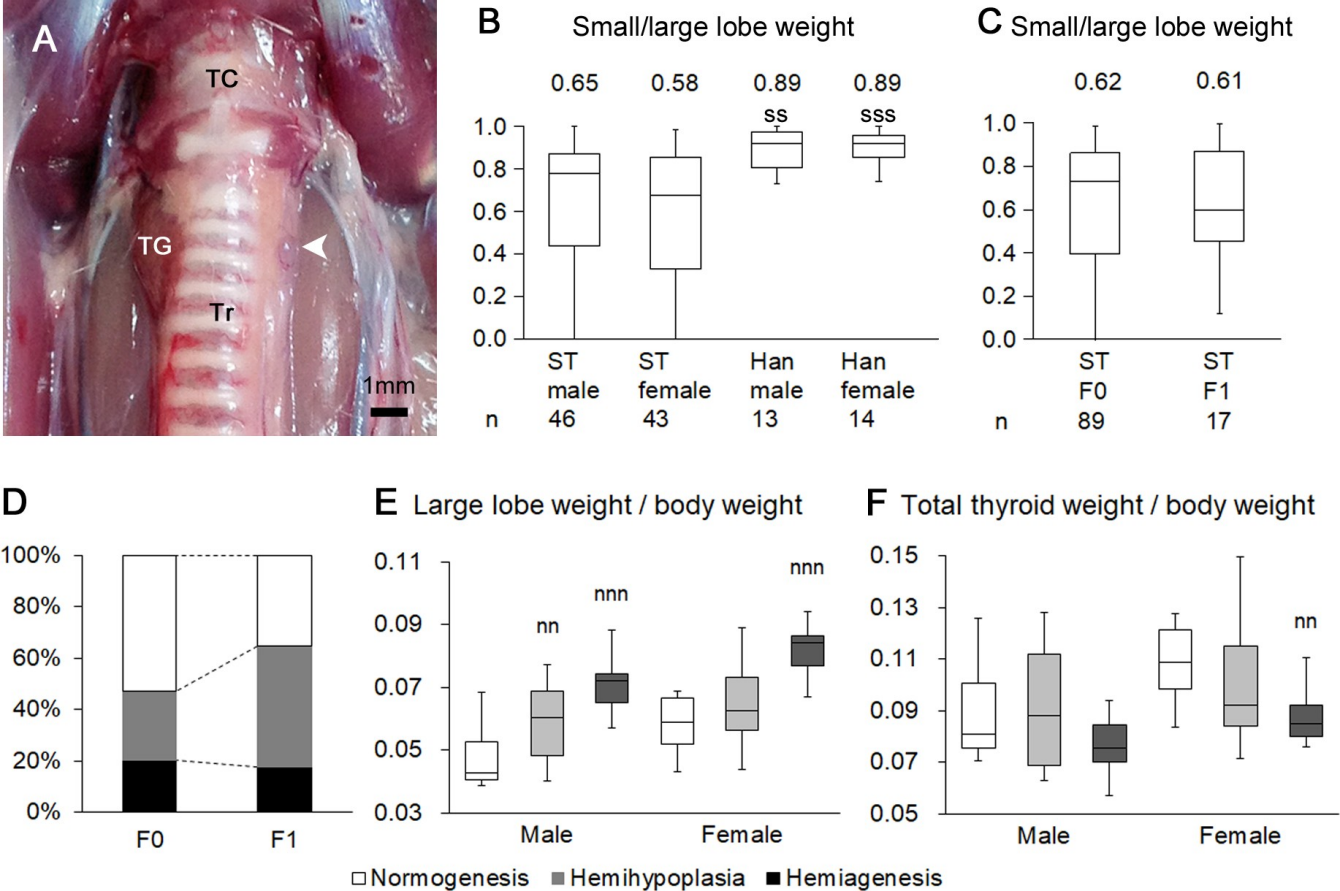
Unilateral thyroid dysgenesis in Wistar/ST rats. (A) Gross features of the thyroid glands in Wistar/ST rats. In this case, the left lobe and isthmus of the thyroid gland is absent, and the parathyroid glands are localized alongside the trachea (arrowhead). TC: thyroid cartilage, TG: thyroid glands, Tr: trachea. (B and C) Ratio of small to large lobe weights of the thyroid glands compared among different rat strains (B) and between F0 and F1 litters from Wistar/ST rats with thyroid hemiagenesis (C). Mean values are expressed above the box plots. ST: Wistar/ST, Han: BrlHan:WIST@Jcl (GALAS). A significant difference from Wistar/ST rats of the same sex is indicated by ss *P* < 0.01 and sss *P* < 0.001, Mann-Whitney *U* test. (D) Prevalence of unilateral thyroid dysgenesis compared between F0 and F1 litters from Wistar/ST rats with thyroid hemiagenesis. (E and F) Relative weight of the large thyroid lobe (E) and that of the total thyroid glands (F) in Wistar/ST rats at 9 to 22 weeks of age. A significant difference from the control group at the same age is indicated by nn *P* < 0.01 and nnn *P* < 0.001, Kruskal-Wallis test followed by Scheffé’s method.

In Wistar/ST rats, each thyroid lobe was frequently different in size, and the thyroid lobe was unilaterally absent in some cases (Fig 1A). On the side lacking the thyroid lobe, the parathyroid glands were normally localized (Fig 1A). We compared the ratio of small to large thyroid lobe weight between Wistar/ST and BrlHan:WIST@Jcl (GALAS) rats to examine strain differences (Fig 1B). The ratio of small to large thyroid lobe weight was higher than 0.75, and 95% confidence interval of the lobe weight ratio was approximately 0.7 in both sexes of BrlHan:WIST@Jcl (GALAS) rats, while the values were highly variable and significantly lower in both sexes of Wistar/ST rats (Fig 1B). In Wistar/ST rats showing 0.3 of the lobe weight ratio, the isthmus of the thyroid gland was visible but the small lobe did not extend toward both the upper and lower poles. Based on the results of the lobe weight ratio, we classified the unilateral thyroid dysgenesis of Wistar/ST rats as follows: rats showing a ratio of small to large lobe weights higher than 0.7 served as the control group and were classified as normogenesis; the lobe weight ratio between 0.3 and 0.7 were classified as hemihypoplasia, with lower than 0.3 classified as hemiagenesis.

Table 3 shows the prevalence of thyroid dysgenesis in Wistar/ST rats. In Wistar/ST rats, 55.8% of females and 39.1% of males presented with either thyroid hemihypoplasia or hemiagenesis, and the female-to-male ratio was 1.33. Moreover, thyroid hemiagenesis tended to occur frequently in female Wistar/ST rats, and the female-to-male ratio was 1.57, although sex-related differences were not found. Complete loss of both the unilateral thyroid lobe and the isthmus were found in 4 males (8.6%) and 6 females (14.0%). Although hemiagenesis was more prevalent on the left side in both sexes, right-sided dysgenesis if present was more often observed in females (N = 6) than in males (N = 1). The prevalence of thyroid dysgenesis did not increase age-dependently in Wistar/ST rats (Table 4).

**Table 3:** Prevalence of thyroid dysgenesis according to sex and laterality.

	Total	Female	Male	Female: male ratio	*P-*value
Sample size	89	43	46		
Thyroid dysgenesis	42 (47.2%)	24 (55.8%)	18 (39.1%)	1.33	*P* = 0.140
Hemihypoplasia	24 (27.0%)	13 (30.2%)	11 (23.9%)	1.18	*P* = 0.634
Hemiagenesis	18 (20.2%)	11 (25.6%)	7 (15.2%)	1.57	*P* = 0.293
Side of presentation					
Left	35 (39.3%)	18 (41.9%)	17 (37.0%)	1.06	*P* = 0.669
Right	7 (7.9%)	6 (14.0%)	1 (1.1%)	6.00	*P* = 0.053

Thyroid dysgenesis represents hemihypoplasia + hemiagenesis. The side of presentation is examined for the rats with thyroid dysgenesis.

**Table 4:** Age-related changes of prevalence of thyroid dysgenesis.

	Total	9–15 weeks	16–22 weeks	P-value
Sample size	89	51	38	
Thyroid dysgenesis	42 (47.2%)	25 (49.0%)	17 (44.8%)	*P* = 0.830
Hemihypoplasia	24 (27.0%)	12 (23.5%)	12 (31.6%)	*P* = 0.472
Hemiagenesis	18 (20.2%)	13 (25.5%)	5 (13.2%)	*P* = 0.188

To estimate the genetic inheritance of thyroid dysgenesis, two pairs of Wistar/ST rats with thyroid hemiagenesis were mated, and the ratio of small to large lobe weight was compared between F0 and F1 litters. In the F1 generation, the average litter size was 8.5 per pair, and no rats died until the sampling. The prevalence of thyroid dysgenesis was 6/10 (60%) in pair #1, 5/7 (71%) in pair #2, and 11/17 (65%) in total. Although median values of the ratio of small to large thyroid lobe weight were lower in F1 litters than in F0, no significant differences were noted (Fig 1C). Also, F1 litters tended to have a higher prevalence of unilateral thyroid dysgenesis, with no significant differences noted when compared to F0 (Fig 1D).

### Physiological and hormonal status in Wistar/ST rats

**Fig 2:**
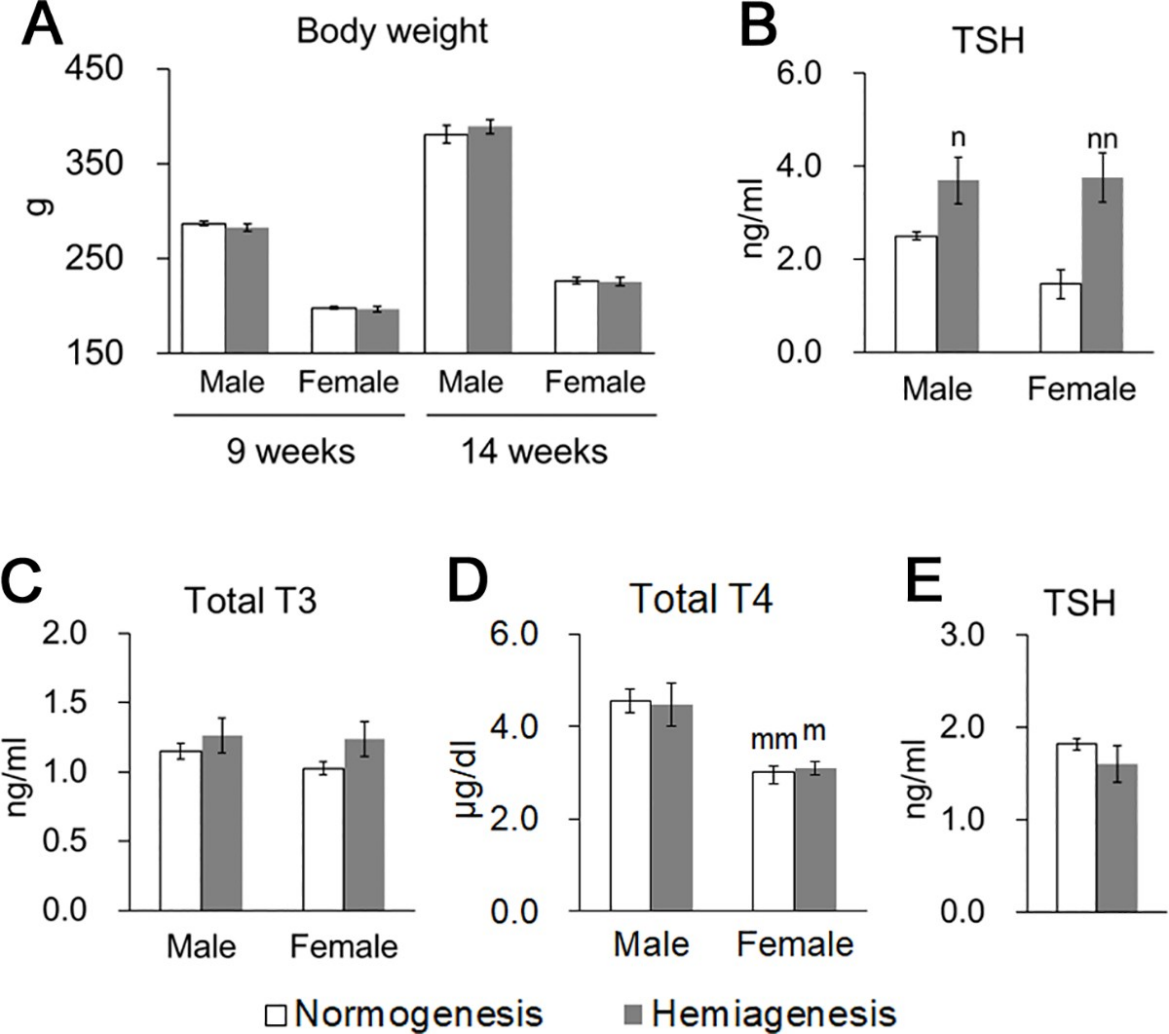
Physiological and hormonal status in Wistar/ST rats. (A) Body weight at 9 and 14 weeks of age. (B-D) Plasma levels of TSH (B), total T3 (C), and total T4 (D) at 7 to 14 weeks of age. (E) Plasma levels of TSH at 22 to 42 weeks of age. At panel E, the control group consists of 4 males and 12 females, and the hemiagenesis group consists of 2 males and 6 females. A significant difference from the control rats of the same sex is indicated as n *P* < 0.05 and nn *P* < 0.01, Mann-Whitney *U* test. A significant sex difference is indicated as m *P* < 0.05 and mm *P* < 0.01, Mann-Whitney *U* test.

Between the ages of 9 and 22 weeks, Wistar/ST rats presenting thyroid hemiagenesis had a higher relative weight of the large lobe than the control rats with thyroid normogenesis in both sexes (Fig 1E). The relative weight of total thyroid glands was lower in rats with thyroid hemiagenesis than in the control female rats, while no significant differences were found in males (Fig 1F). These results indicated that the weight of the hemiagenetic thyroid lobe increased in both sexes and reached the total thyroid weight of the control rats in male Wistar/ST rats. Body weight of Wistar/ST rats with thyroid hemiagenesis was similar to that of the control Wistar/ST rats in both sexes at 9 and 14 weeks of age (Fig 2A). In the control rats aged 7 to 14 weeks, plasma total T4 levels were significantly higher in males than in females as previously reported [16], while no sex-related differences were found in TSH and total T3 levels (Fig 2B–2D). In both sexes, rats presenting thyroid hemiagenesis showed higher plasma TSH levels than the control rats at 7 to 14 weeks of age, but plasma TSH levels became similar at 22 to 42 weeks of age (Fig 2B and 2E). In contrast, total T3 and T4 levels in the plasma did not differ between rats with or without thyroid hemiagenesis in both sexes (Fig 2C and 2D).

### Histopathological examination of the thyroid glands

**Fig 3:**
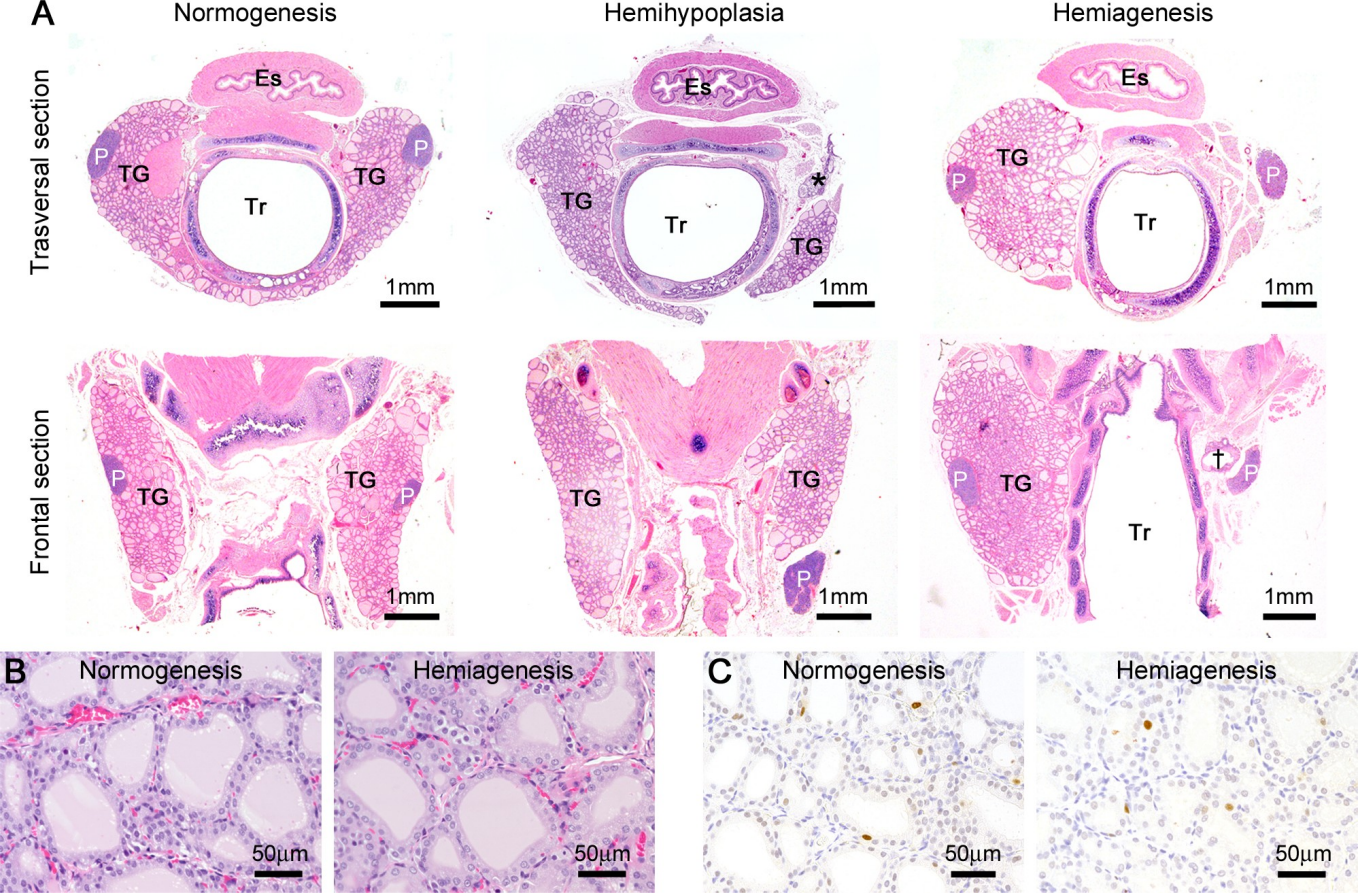
Histopathological features of thyroid glands in Wistar/ST rats. (A) Transversal and frontal section of the thyroid glands. Es: esophagus, P: parathyroid gland, TG: thyroid gland, Tr: trachea. Asterisk and dagger indicate nodule and cystic structures, respectively. (B and C) HE section (B) and immunohistochemistry for PCNA (C) in the central region of the thyroid glands at 9 to 14 weeks of age.

We histopathologically examined the thyroid glands in Wistar/ST rats. Although Wistar/ST rats represented unilateral thyroid dysgenesis and the remaining lobes increased in weight, the thyroid glands showed no abnormalities such as cellular infiltration and goiter (Fig 3A). The follicular epithelial cells neither increased in height nor showed active proliferating activities in rats with thyroid dysgenesis in both sexes at 9 to 14 weeks old (Fig 3B and 3C). In normal Wistar/ST rats, the parathyroid glands were embedded in the thyroid glands, and they tended to be localized at the level of the isthmus (Fig 3A). In some instances, especially in cases with thyroid hemihypoplasia, the parathyroid glands were not embedded in the thyroid lobes (Fig 3A). At the side of the hemiagenetic thyroid lobe, the parathyroid glands tended to be localized at the same position as the contralateral glands (Fig 3A). Although the localization of the parathyroid glands was variable, their size was normal in all cases (Fig 3A).

**Fig 4:**
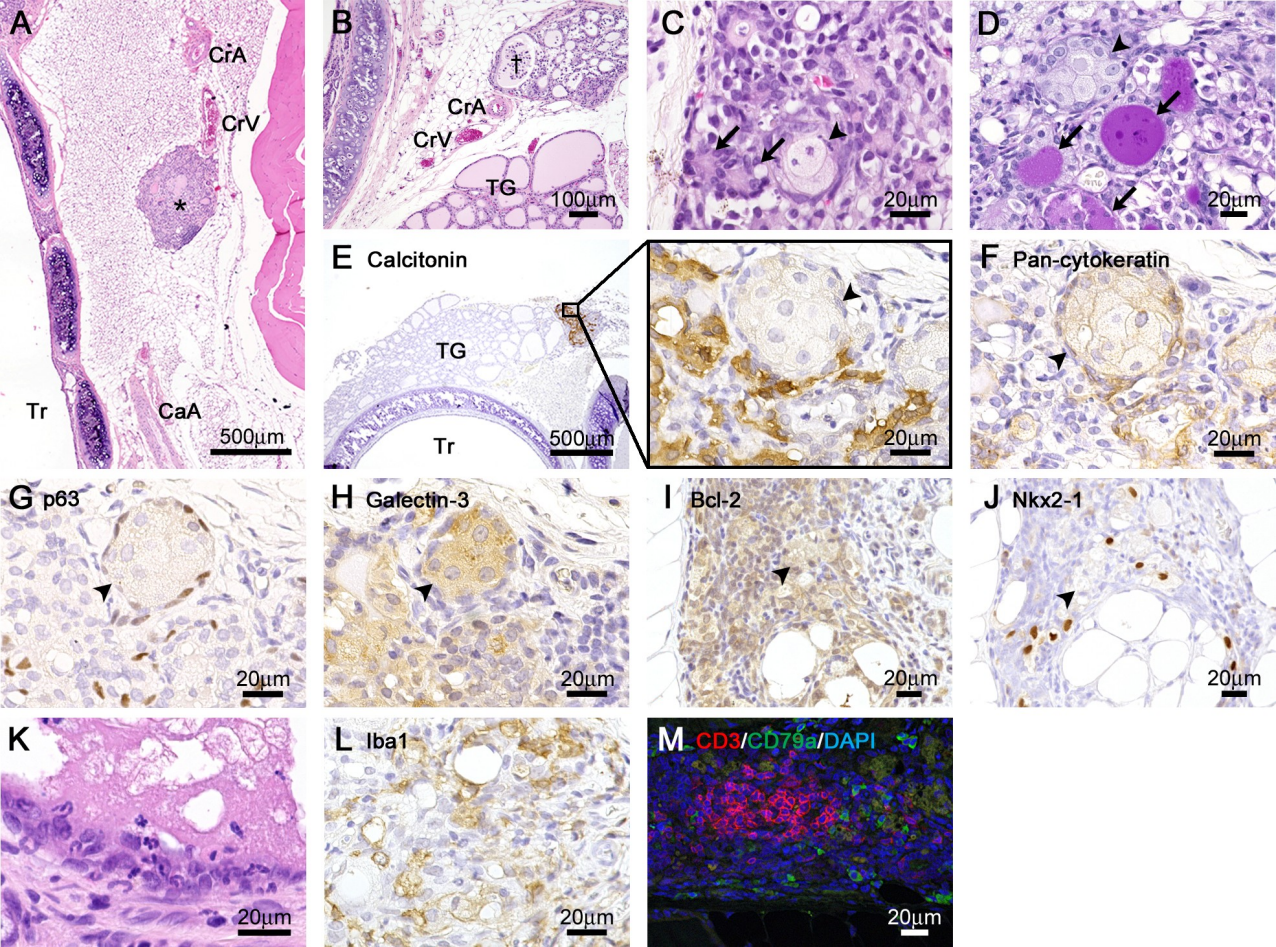
Histopathological features of the nodules and cystic structures in Wistar/ST rats. (A and B) Localization of the blood vessels at the side of hemiagenesis (A) and hemihypoplasia (B). (C and D) Higher magnification of the nodules. HE sections (C) and PAS section (D). The nodules contain PAS-positive follicular structures and cell nests. (E-J) Immunohistochemistry of the nodules for calcitonin (E), pan-cytokeratin (F), p63 (G), galectin-3 (H), Bcl-2 (I), and Nkx2-1 (J). (K-M) Immune cell infiltration into the nodules. HE sections (K), immunohistochemistry for Iba1 (L), and immunofluorescence for CD3 and CD79a (M). CaA: caudal thyroid artery, CrA: cranial thyroid artery, CrV: cranial thyroid vein, TG: thyroid gland, Tr: trachea. Asterisks and daggers indicate nodules and cystic structures, respectively. Arrows and arrowheads indicate follicular structures and cell nests, respectively.

Notably, nodules or cystic structures were found beside the dorsal region of the cranial pole of the thyroid lobes in some cases of thyroid hemihypoplasia (Fig 3A) and alongside the trachea in cases of thyroid hemiagenesis (Fig 3A). Both of the nodules and cystic structures always occurred at the side of thyroid dysgenesis, ranging from the cranial pole to the level of the isthmus (Fig 3A), and were localized adjacent to the cranial thyroid artery and vein (Fig 4A and 4B). On the side of the hemiagenesis, the caudal thyroid artery was also present (Fig 4A). The nodules and the cystic structures contained PAS-positive follicular structures and cell nests (Fig 4C and 4D). Calcitonin-positive C-cells distributed in the nodules and cystic structures but not to the adjacent thyroid lobe (Fig 4E). The cell nests were positive for pan-cytokeratin, p63, galectin-3, and Bcl-2 but negative for Nkx2-1 and calcitonin (Fig 4E–4J). Neutrophils, Iba1-positive macrophages, CD3-positive T-cells, and CD79a-positive B-cells infiltrated into the nodules and the cystic structures in all cases (Fig 4K–4M). The lymphocytes formed neither germinal centers nor cortex/medulla structures (Fig 4M).

## Discussion

This study showed that Slc:Wistar/ST rats developed unilateral thyroid dysgenesis that shared several similarities with thyroid hemiagenesis in humans. First, thyroid dysgenesis occurred mainly on the left side and showed no sex differences, while right-sided thyroid dysgenesis tended to be predominant in females in both Wistar/ST rats and humans [1, 8, 17]. Second, thyroid hemiagenesis neither altered body weight nor induced histopathological abnormalities in the thyroid glands in Wistar/ST rats, indicating that thyroid hemiagenesis did not lead to clinical symptoms [1]. Third, Wistar/ST rats with thyroid hemiagenesis showed high levels of TSH levels at a young age, but not in mature adults. Moreover, in human patients with thyroid hemiagenesis, serum TSH levels are significantly higher than in control patients [12]. Fourth, the remaining thyroid lobe became enlarged in both sexes in Wistar/ST rats with normal total T3 and T4 levels, indicating euthyroidism. In humans presenting with thyroid hemiagenesis, the prevalence of compensatory growth of the remaining thyroid lobe increased age-dependently with normal free T4 levels, although few cases of overt hypothyroidism have also been reported [1, 12]. Importantly, the prevalence of thyroid hemiagenesis where the lobe weight ratio was lower than 0.3 was approximately 15% in males and 26% in females of Wistar/ST rats, which was considerably higher than in humans where the prevalence rate is between 0.05% and 0.2% [1]. In Wistar/ST rats, the prevalence of true hemiagenesis, which lacks the isthmus, was 4 out of 46 cases in males (8.6%) and 6 out of 43 cases in females (14.0%), which was about half of the cases of hemiagenesis under these experimental criteria. This ratio was consistent with human cases [18]. When considered together, these results show that Wistar/ST rats might be useful animal models for thyroid hemiagenesis in humans.

At 7 to 14 weeks, Wistar/ST rats lacking one thyroid lobe showed temporal elevation of plasma TSH and normal levels of total T3 and T4, while rats with hemithyroidectomy showed sustained elevation of TSH levels and a slight decline of total T3 and T4 levels [19]. In Wistar/ST rats, the remaining thyroid lobe increased in weight, but the follicular epithelial cells appeared normal in both height and proliferating activities. In addition, embryonic thyroid glands develop independently from systemic regulation by TSH in mice, but TSH has a mitogenic effect on thyroid follicular cells after birth [20]. Although TSH levels were not measured before 7 weeks of age, these results indicate that enlargement of the thyroid lobes actively occurred postnatally and euthyroidism prevailed at 7 to 14 weeks of age. After birth, the hypoplasitic lobes did not show age-related unilateral regression, indicating that the unilateral dysgenesis occurred before birth and “thyroid hemiagenesis,” meaning a lack of one thyroid lobe during embryogenesis, might be an appropriate term for the Wistar/ST rats.

Several candidate genes for thyroid hemiagenesis have been identified in mice, such as *Frs2α*, *Hoxa3*, *Pax3*, *Shh*, and *Tbx1* [1]. *Nkx2-1* and *Pax8* double heterozygous mice of 129/Sv genetic background display thyroid hemiagenesis and hypothyroidism, indicating a polygenic origin of the thyroid hemiagenesis [21]. In *Shh* knockout mice, the thyroid glands fail to form the bilobulated glands and localized to the left side of the trachea; however, *Shh* mRNA is not expressed in the thyroid precursor cells [22]. Knockout mice for *Tbx1*, a downstream target of *Shh*, also fail to form symmetric thyroid lobes, although the remaining thyroid glands are hypoplastic and lack C-cells [23]. Interestingly, thyrocytes produce thyroglobulin in both *Shh*- and *Tbx1*-knockout mice [22, 23]. These results indicate that the thyroid morphogenesis is controlled by both cell-autonomously and non-cell-autonomously. While in humans with thyroid hemiagenesis, no mutations are found in *PAX8*, *NKX2-1*, *FOXE1*, *HHEX*, *TBX1*, and *SHH* genes, indicating other responsible genes are present in humans [24]. In the present study, unilateral thyroid dysgenesis was strain-dependent in rats, indicating the genetic effect. Although Wistar/ST rats developed thyroid hemiagenesis, the rats exhibited euthyroidism and calcitonin-positive C-cells were present, which resembles the clinical features in humans [1]. Therefore, Wistar/ST rats might carry novel genes responsible for thyroid hemiagenesis. The F1 litters between Wistar/ST rats with thyroid hemiagenesis inherited thyroid dysgenesis in a similar prevalence to F0. If thyroid dysgenesis had been controlled only through genetic factors, the prevalence might have been higher in F1 litters than in F0 generation rats under this experimental condition. These results indicate that unilateral thyroid dysgenesis is also affected by sporadic factors in Wistar/ST rats. To directly elucidate the influence of the genetic background, the creation of an inbred strain is required, as Wistar/ST rats are an outbred strain.

The morphogenesis of the thyroid gland is strongly dependent on the cardiovascular system [1, 25]. The thyroid primordium descends along with the aortic sac during embryogenesis [25]. The thyroid primordium bilobulates parallel to the blood vessels of the third pharyngeal arches, and thyroid hemiagenesis is considered to be caused by bilobulation defects of the thyroid primordium [1, 25]. In addition, embryonic thyroid cells recruit microvessel ingrowth that reciprocally stimulates further growth of the thyroid primordium and folliculogenesis [26]. In Wistar/ST rats, although the final position of the thyroid gland was disturbed, other abnormalities affecting thyroid functions were not found. Taken together, it is hypothesized that the extrinsic factors might affect the thyroid morphogenesis rather than thyroid primordium itself, and the embryonic large vessels are one of the candidates for the thyroid dysgenesis in Wistar/ST rats. One recent study demonstrated that thyroid arteries were absent on the ipsilateral side of the thyroid hemiagenesis in humans, indicating that inadequate blood supply also leads to aplasia of the thyroid lobe during embryogenesis [27]. In the Wistar/ST rats, both cranial and caudal thyroid arteries were found at the side of the thyroid hemiagenesis, indicating that the thyroid hemiagenesis was unrelated to a defect in blood supply. Therefore, to clarify the pathogenesis of thyroid dysgenesis, histopathology of the thyroid glands in fetuses and neonates will be required in future.

Our results also shed light on the morphogenesis of pharyngeal pouch-derived organs. In wild-type mice at embryonic day 13.5 before the ultimobranchial body fuses with the thyroid primordium, the ultimobranchial body is composed of two types of cells: one expressing Nkx2-1 and the other expressing p63, and both markers do not overlap each other [28]. The ultimobranchial body remnants are normally found in the thyroid gland, namely solid cell nests, which express p63, cytokeratins, and galectin-3 and are frequently surrounded by Nkx2-1 expressing C-cells [14]. The nodules or cystic structures found in Wistar/ST rats included calcitonin-positive C-cells and cell nests positive for the ultimobranchial body markers. These results strongly indicated the nodules or cystic structures were ultimobranchial body remnants. In addition, the ultimobranchial body remnants contained PAS-positive follicular structures, indicating an ultimobranchial origin for some follicular epithelial cells in humans [29]. In Wistar/ST rats, inflammatory cells accumulated into the ultimobranchial body remnants. Since the lymphocytes neither formed germinal centers nor cortex/medulla structures, they were unrelated to the lymph nodes and ectopic thymi. It is possible that the inflammatory cells contribute to the pathogenesis of the ultimobranchial body remnants via the proliferation of the epithelial component, as the piriform sinus fistula originating from the ultimobranchial body shows chronic inflammation and their caudal end in the thyroid gland actively proliferate to form branched structures in cotton rats [30, 31].

**Fig 5:**
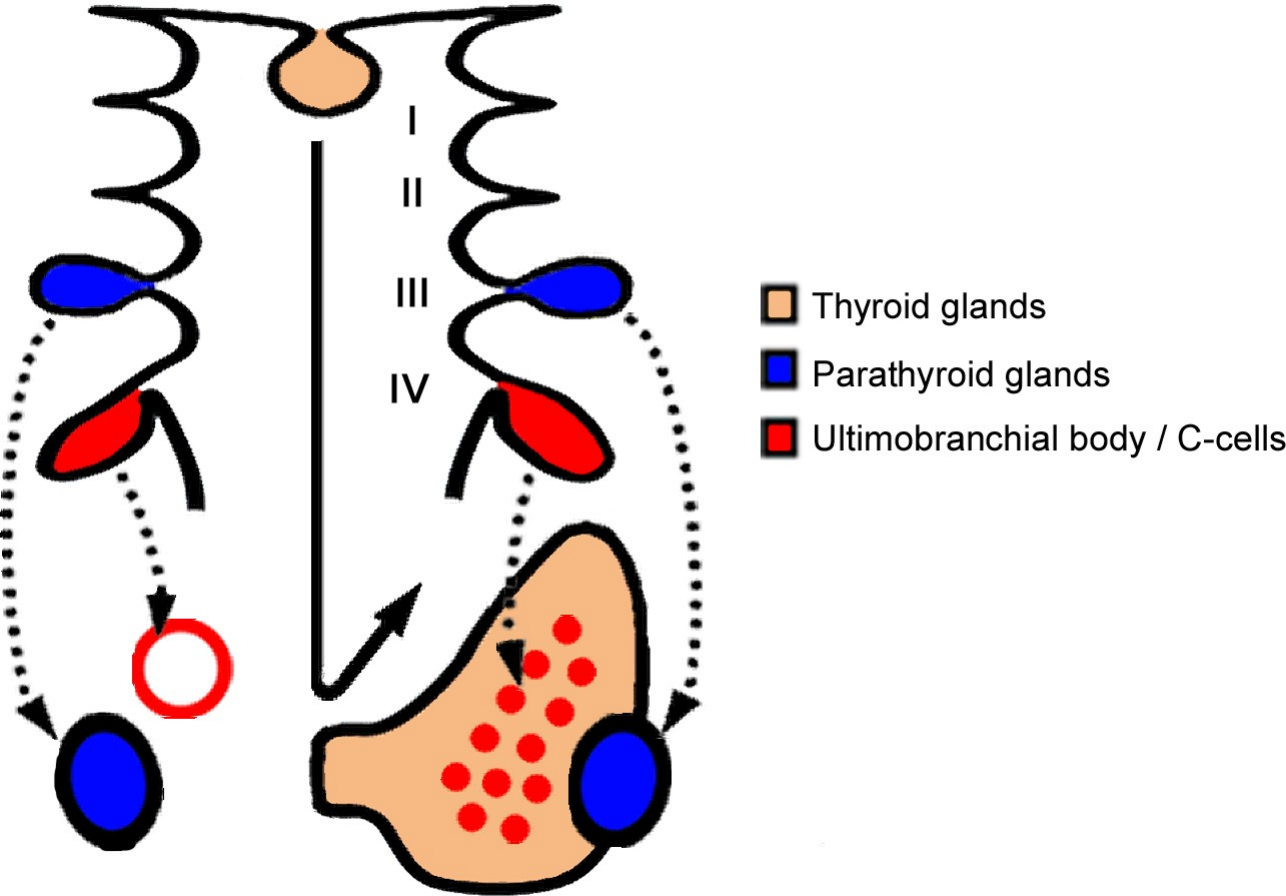
Distribution of the laryngeal endocrine organs in Wistar/ST rats. The right side represents normal development, and the left side shows thyroid hemiagenesis. Numbers I to IV indicate the number of pharyngeal pouches.

Localization of the laryngeal endocrine organs of Wistar/ST rats is summarized in Fig 5. In Wistar/ST rats with thyroid normogenesis, C-cells are included within the bilobulated thyroid glands. The parathyroid glands were embedded within the thyroid glands, but they were separated by connective tissues, as reported previously [3]. In Wistar/ST rats lacking the unilateral thyroid lobe, the parathyroid glands had migrated to their normal locations and showed normal size, indicating that the thyroid glands were not required for the migration of the inferior parathyroid glands in rats. In cases of thyroid dysgenesis, C-cells were found as the ultimobranchial body remnants around the thyroid glands, and were not found in the adjacent thyroid lobe, indicating loss of fusion. Similar phenomena have been found in dogs and one-humped camels, namely C-cell complexes, which are the persistent ultimobranchial bodies that have incompletely fused with the thyroid gland [3, 32]. These results indicate that the thyroid gland is not required for the migration of the ultimobranchial body, although loss of fusion between the ultimobranchial body and the thyroid gland leads to the formation of the ultimobranchial body remnants. Notably, the ultimobranchial body remnants were localized adjacent to the cranial thyroid artery and vein. In other animal species, the ultimobranchial body descends to the thyroid gland along with the blood vessels in mice [33], ultimobranchial body remnants were localized around the entry of the cranial thyroid artery and vein in one-humped camels [32], and the piriform sinus fistula originating from the ultimobranchial body runs along with the cranial thyroid artery in cotton rats [30]. This study further supports the hypothesis that the ultimobranchial body is also dependent on the cardiovascular system for its migration and enters the thyroid gland along the cranial thyroid artery or vein.

In conclusion, Wistar/ST rats developed unilateral thyroid dysgenesis and appear to be novel and useful animal models for thyroid hemiagenesis in humans. This strain might also be useful for understanding mechanisms of migration with regard to pharyngeal pouch-derived organs such as the parathyroid glands and the ultimobranchial body.
